# EP37 A need to train paediatric rheumatologists in musculoskeletal ultrasound scanning. How do we move forward?

**DOI:** 10.1093/rap/rkaa052.036

**Published:** 2020-11-03

**Authors:** Fatemeh Jenabi, Katie Mageean, Alice Leahy, Brian Davidson, Hans de Graaf

**Affiliations:** University Hospital of Southampton, Southampton, UK

## Abstract

**Case report – Introduction:**

Musculoskeletal ultrasound is used by clinicians around the world and learning this skill is included in paediatric rheumatology training programmes in several countries. However, in the UK only a few clinicians use it in their daily practice. British Society of Rheumatologists has recently shown interest in ultrasound scan training in paediatric rheumatology. Paediatric rheumatologist team in Wessex would like to set up an ultrasound training module for paediatric rheumatology for anyone interested, including trainees and consultants. The team aimed to check the clinicians' interest and demand for it nationally.

**Case report - Case description:**

A brief questionnaire was sent to 45 paediatric rheumatologist consultants in the UK and 14 paediatric rheumatology trainees to gain more information about the use of MSK-USS in clinic. We also sought the clinicians' opinion to ensure the potential ultrasound scan module will meet their needs. 40 out of 45 paediatric rheumatologists replied (response rate of 89%) and 7out of 14 specialist trainees responded (response rate 50%). 80% (32) consultants and all paediatric rheumatology trainees felt that musculoskeletal ultrasound (MSK-USS) performed by a clinician in clinic would benefit their patients. Majority stated that for urgent cases, it could take up to 2 weeks in their centre for a departmental USS to be done and reported. Only 32.5% (13) could arrange MSK- USS on the same day for urgent scans. The number of MSK-USS and MRI scans requested per month were similar. 70% (28) of the clinicians and trainees have access to an ultrasound scanner. Majority of clinicians expressed their enthusiasm (median of 80%) for an interactive paediatric rheumatology musculoskeletal ultrasound online module as well as the platform in which images and clips. 100% (7) of trainees were keen to learn MSK-USS as part of their training and majority felt that they could dedicate regular time for it alongside their other clinical duties.

**Case report - Discussion:**

This study highlighted that various paediatric rheumatology departments within the UK already had discussions about the use of MSK-USS as part of clinical practice without making progress.

Majority of paediatric consultants in the UK feel that USS performed by the clinician is beneficial for the patients, particularly for image guided injections and performing synovial biopsies. However, a small group reported reservations due to inter-operator variation and challenges of interpreting non classical signs on scan as well as the risk of over-interpretation of scan findings regarding inflammation. Moreover, another obstructing factor for some consultants to use MSK-USS can be time constraints in terms of becoming proficient in MSK-USS and time to perform USS in the clinic.

**Case report - Key learning points:**

This study highlighted that various paediatric rheumatology departments within the UK already had discussions about the use of MSK-USS as part of clinical practice without making progress. Majority of paediatric consultants in the UK feel that USS performed by the clinician is beneficial for the patients, particularly for image guided injections and performing synovial biopsies. However, a small group reported reservations.

Wessex rheumatology team is in the process of setting up an ultrasound training module for paediatric rheumatology which could be what is needed to push discussion into action. We intend to carry out the same study in other European countries such as Italy, Netherlands, France and Germany to gather more evidence. Given the lack of evidence in this area, such studies would be important in shaping the future clinical practice of paediatric rheumatology.

Taking the high interest rate of current trainees, we also recommend addition of a specific ultrasound training module for paediatric rheumatology trainees as part of the GRID (specialist) curriculum.

